# Supplementary material for: Clot formation, structure, and fibrinolysis of plasma from pancreatic cancer patients
Source: J Thromb Thrombolysis. 2025 Jul 14;58(8):1058–70. doi: 10.1007/s11239-025-03118-x (PMC12740958; doi:10.1007/s11239-025-03118-x)
Supplement: Supplementary file 1 — Supplementary file1 (DOCX 415 KB) [file 11239_2025_3118_MOESM1_ESM.docx]

**SUPPLEMENT**

1. Select lab results.

*Supplemental Table 1. Presence of cells. Red fill indicates out of the healthy range. Green fill indicates within the healthy range. Complete blood count (CBC).*

| Cell type | Mean ± standard error of the mean | Normal CBC [36] |
| --- | --- | --- |
| Hematocrit (%) | 34.99 ± 0.9560 | Male: 40-54%  Female: 36-48% |
| Red blood cell count (10*6/uL) | 3.792 ± 0.1280 | Male: 4.6-6.2  Female: 4.2-5.4 |
| White blood cells (10*3/uL) | 14.91 ± 1.680 | 4.5 - 11 |
| Platelets (10*3/uL) | 208.1 ± 11.28 | 150-400 |

*Supplemental Table 2. Liver function tests. Red fill indicates out of the healthy range. Green fill indicates within the healthy range. Complete blood count (CBC).*

| Liver Function Tests | Mean ± standard error of the mean | Normal CBC |
| --- | --- | --- |
| Bilirubin (mg/dL) | 0.9100 ± 0.2042 | 0.1-1.2 |
| **AST (Aspartate Aminotransferase) (U/L)** | 111.7 ± 24.95 | 8 - 33 |
| ALT (Alanine Aminotransferase) (U/mL) | 159.7± 57.99 | 10-50 |
| **Alkaline Phosphatase (ALK PHOS) (U/mL)** | 73.10±15.13 | 45-115 |
| Albumin (g/dL) | 3.359 ± 0.1015 | 3.5-5.5 |

*Supplemental Table 3.* Metabolic and inflammatory markers*. Red fill indicates out of the healthy range. Green fill indicates within the healthy range. Complete blood count (CBC).*

| Metabolic and inflammatory markers | Mean ± standard error of the mean | Normal CBC |
| --- | --- | --- |
| **Amylase (U/mL)** | 159.9 ± 108.6 | 28-100 |
| **CRP (C-Reactive Protein) (mg/dL)** | 3.370 ± 0.6966 | <0.3 |
| Glucose (mg/dL) | 176.3 ± 12.12 | 70-100 |

The above parameters (Supplemental Tables 1-3) highlight abnormal cell counts, poor liver function, and elevated metabolic and inflammatory markers in our PC patient cohort. While hematocrit and RBC count are decreased and WBC count is increased compared to normal levels, platelets were within the healthy range. Only one patient (out of 17) was denoted to have low platelet levels (Supplemental Table 1). We then determined the liver function of PC patients and identified values within the normal range for bilirubin and alkaline phosphatase. However, there are elevated levels of **aspartate aminotransferase and** alanine aminotransferase as well as reduced albumin (Supplementary Table 2). Lastly, there are elevated metabolic and inflammatory markers (amylase, C-reactive protein, and glucose, Supplemental Table 3). Despite many irregular factors, none of them correlate to PAI-1 concentration (Supplemental Figure 5).

2. Activation of clotting with TF (rather than phospholipids)

In the main text we used phospholipids and calcium chloride to initiate clotting; however, TF (1 pM) can also be used to initiate clotting. We show that there is a correlation between an increase in max OD for both TF and phospholipids (Supplemental Figure 1a, p=0.0001), but phospholipids were able to generate clots with an overall higher max OD (Supplemental Figure 1b). There was not a correlation between the two activators for lag time (Supplemental Figure 1c, ns). It took much longer to initiate clotting with phospholipids compared to TF (Supplemental Figure 1d). There was neither a correlation nor a difference in time to 50% between the two activators (Supplemental Figure 1e, ns, f, ns).

*Supplemental Figure 1. Clotting and fibrinolysis with TF activation. Comparison of max OD (a, b), clotting lag time (c, d), and time to 50% lysis (e, f). **** p<0.0001.*

1. Comparisons between time to 50% lysis, degradation rate, and lag time.

The more time it took to break down (delayed time to 50% lysis), the slower it was degrading (slower degradation rate) (Supplemental Figure 2a, p<0.0001). There was a correlation between clotting lag time and time to 50% lysis as well as degradation for PC patients, but not healthy donors. The delayed and slower fibrinolysis were patients that had delayed initiation of clotting (Supplemental Figure 2b, c, p<0.05). PTT correlated to time to 50% lysis (Supplemental Figure 2d, p<0.05).

**

*Supplemental Figure 2. Role of degradation. Comparison between a) time to 50% lysis and degradation rate, b) clotting lag time and time to 50% lysis, and d) clotting lag time and degradation rate. d) Comparison between PTT and time to 50% lysis. Pink was PC, black was healthy donor.*

1. PTT and anticoagulants

Prolonged PTT cannot be attributed to anticoagulants given as there is not a trend in common treatments as seen in Supplemental Table 4.

*Supplemental Table 4. PTT and anticoagulants given to individual patients.*

| PTT (seconds) | Anticoagulants |
| --- | --- |
| 28.55 | heparin, enoxaparin |
| 32 | heparin |
| 25.2 | Apixaban, heparin |
| 26.2 | heparin |

1. Clot structure and clotting kinetics

For PC patients, fibrin network pore size does not correlate to rate of formation, time to 50% lysis or max OD (Supplemental Figure 3a-c, ns). Type of anticoagulant does not alter pore size (Supplementary Figure 3d). An increase in fiber diameter correlated to a higher maximum optical density (Supplemental Figure 3d, p<0.01), but not the rate of formation (Supplemental Figure 3f, ns). Diameter does not correlate to time to 50% lysis (Supplemental Figure 3g, ns). A longer PTT correlates to a thicker fiber diameter (Supplemental Figure 3h, p<0.05).

**

*Supplemental Figure 3. Fibrin network structure relationships. Relationship between fibrin network pore size and a) rate of formation, b) time to 50% lysis, c) max OD, and d) anticoagulants. Relationship between fiber diameter and e) rate of formation, f) max OD, g) time to 50% lysis, and h) PTT. Pink was PC, black was healthy donor.*

1. Comparisons with factor levels.

Neither thrombin generation nor fibrinogen concentration correlated to clotting lag time (Supplemental Figure 4a, ns, b, ns). An increased fibrinogen concentration correlated to a decreased hematocrit, increased max OD, and increased fiber diameter (Supplemental Figure 4c, p<0.05, d, p<0.0001, e, p<0.05). Neither thrombin generation nor fibrinogen concentration correlated to time to 50% lysis (Supplemental Figure 4f, ns, g, ns). An increased concentration of PAI-1 correlated to a delayed time to 50% lysis and a delayed partial thrombin time (PTT) (Supplemental Figure 4h, p<0.01, i, p<0.05). There was not a correlation between TAFI and time to 50% lysis (Supplemental Figure 4j, ns).

*Supplemental Figure 4. Relationships with clotting and fibrinolytic factors. a) Relationship between thrombin generation and clotting lag time. Relationships between fibrinogen concentration and c) clotting lag time, c) hematocrit, d) max OD, and e) diameter. Relationship between f) fibrinogen concentration and g) thrombin generation with time to 50% lysis. Relationship between PAI-1 and h) time to 50% lysis and I) PTT. j) Relationship between TAFI and time to 50% lysis. Pink was PC, black was healthy donor.*

1. Comparing cell counts and other lab parameters to PAI-1 concentrations.

There was no correlation (ns) between any cell counts or lab denoted in Supplemental Tables 1-3 parameters and PAI-1 concentrations (Supplemental Figure 5).

**

*Supplemental Table 5. Plots detailing the relationships between lab parameters and PAI-1 concentrations.*
